# Sleep Spindle Characteristics in Children with Neurodevelopmental Disorders and Their Relation to Cognition

**DOI:** 10.1155/2016/4724792

**Published:** 2016-07-11

**Authors:** Reut Gruber, Merrill S. Wise

**Affiliations:** ^1^Department of Psychiatry, McGill University, 6875 LaSalle Boulevard, Montreal, QC, Canada H4H 1R3; ^2^Methodist Healthcare Sleep Disorders Center, 5050 Poplar Avenue, Memphis, TN 38157, USA

## Abstract

Empirical evidence indicates that sleep spindles facilitate neuroplasticity and “off-line” processing during sleep, which supports learning, memory consolidation, and intellectual performance. Children with neurodevelopmental disorders (NDDs) exhibit characteristics that may increase both the risk for and vulnerability to abnormal spindle generation. Despite the high prevalence of sleep problems and cognitive deficits in children with NDD, only a few studies have examined the putative association between spindle characteristics and cognitive function. This paper reviews the literature regarding sleep spindle characteristics in children with NDD and their relation to cognition in light of what is known in typically developing children and based on the available evidence regarding children with NDD. We integrate available data, identify gaps in understanding, and recommend future research directions. Collectively, studies are limited by small sample sizes, heterogeneous populations with multiple comorbidities, and nonstandardized methods for collecting and analyzing findings. These limitations notwithstanding, the evidence suggests that future studies should examine associations between sleep spindle characteristics and cognitive function in children with and without NDD, and preliminary findings raise the intriguing question of whether enhancement or manipulation of sleep spindles could improve sleep-dependent memory and other aspects of cognitive function in this population.

## 1. Introduction

Neurodevelopmental disorders (NDDs) are a heterogeneous group of conditions in which the development of the central nervous system is disrupted. Manifestations can include impairments in motor function, learning, cognition and/or communication, or neuropsychiatric problems. These issues appear early in development, persist throughout life, and produce notable impairments in social, communicative, cognitive, and behavioral functioning [1] that can vary from very specific limitations to global impairment in intelligence and social skills. This group of disorders includes intellectual disability (formerly referred to as mental retardation), communication disorders, autism spectrum disorder (ASD), Attention Deficit Hyperactivity Disorder (ADHD), specific learning disorders, and neurodevelopmental motor disorders including cerebral palsy (CP).

Sleep is a vital process for brain restoration and it is critical for maintaining cognitive function. Strong empirical evidence indicates that sleep spindles facilitate the plasticity which supports learning, memory consolidation, declarative learning, motor skills, and overall intellectual performance. The cognitive functions that are related to sleep spindles are also key domains of dysfunction in children with NDD [2].

Clinically significant sleep problems are prevalent in children and adolescents with NDD [3, 4]. Among children with neurocognitive difficulties, the impact of disrupted sleep spindle generation may be amplified. Hence, the characteristics of NDD may increase both the risk for and vulnerability to abnormal sleep spindle generation. The goal of the present review is to review and integrate existing evidence regarding sleep spindle characteristics in children with NDD and, when evidence is available, to examine the associations between these characteristics and cognitive function.

A better understanding of relationships between sleep and NDD is expected to provide insight into the pathophysiology and possibly the treatment of such disorders and improve our understanding of the association between sleep spindles and cognition in children. It is likely that sleep spindle characteristics represent a marker of brain development and function, as well as a window into underlying brain mechanisms that support cognition.

We will first briefly describe sleep spindle development and its relationship with cognitive development and function in typically developing children. We will then review the available evidence regarding sleep spindle characteristics in children with a variety of NDDs and their relation to cognition. Finally, we will discuss the findings, identify gaps in understanding, and recommend future research directions in this emerging area of investigation.

## 2. Sleep Spindles in Typically Developing Children

Sleep spindles represent an oscillating electrical potential in the brain; they have a characteristic frequency of 11–16 Hz (usually 12–14 Hz in healthy adults) and last from one to several seconds in duration [5]. On scalp electroencephalography (EEG), spindles are seen as sinusoidal waves that often have a fusiform or “crescendo-decrescendo” morphology [6].

Sleep spindles are characterized by their symmetry; synchrony between hemispheres; amplitude, which is the peak-to-peak difference in spindle size, reflecting voltage; frequency, which is the number of waveforms per second; density, which is the number of spindle bursts/min of NREM sleep; and the duration of spindle bursts. In infants, sleep spindles last several seconds in duration, are expressed maximally in the frontocentral location, are in the high alpha or low beta frequency range, and are not synchronous. The lack of synchrony is likely due to lack of myelination in the neonatal brain. By 2 years of age, it is considered abnormal if most spindles are still asynchronous [7]. In older children and adults, sleep spindles are expressed diffusely across the head but maximally over the central regions and in a bilaterally synchronous and symmetric fashion [7]. Moreover, sleep spindles can be divided into two distinct types based on their frequency and field of expression. Slower spindles (9 to <13 Hz) occur maximally over the frontal regions, whereas faster spindles (>13–16 Hz) dominate [8, 9] over the central and parietal head regions and typically precede slow spindles by hundreds of milliseconds [9–11]. The slow spindles display a typical waxing-and-waning pattern, whereas fast spindles are mainly waning [12]. This difference begins to develop at around 2 years of age [13]. The two populations of sleep spindles are thought to arise from and represent different generators within the thalamus, with some level of cortical involvement. They also demonstrate different maturational patterns, suggesting that their development is associated with changes in thalamocortical structures and maturation of the physiological systems that produce spindles [6, 14, 15]. Shinomiya et al. suggested that separate investigation of the two types of spindles may be important in evaluating developmental processes in the central nervous systems of children and adolescents, and that frontal spindle activity could represent a good indicator of biological maturation [15].

### 2.1. Generation of Sleep Spindles

Sleep spindles are a prototypical thalamocortical rhythm generated and “paced” in the thalamus via a network of synaptic interactions involving inhibitory (GABAergic) neurons in the reticular thalamic nuclei, thalamocortical cells, and cortical pyramidal neurons [16]. In animal models, the spindle rhythm is abolished by destruction of the thalamus, whereas it persists after decortication when the thalamus is preserved [14, 17]. Spindle frequency is determined in large part by an interplay between mutually interconnected GABAergic inhibitory neurons of the reticular nucleus of the thalamus and the thalamocortical neurons, with influence from inputs of the cortex and brainstem [14]. Reticular thalamic neurons impose hyperpolarization on thalamocortical neurons, thereby activating a nonspecific cation current that depolarizes the thalamocortical neurons and activates low-threshold calcium ion currents and bursting. The latter process provides feedback excitation to reticular thalamic neurons, thus closing the loop. Each thalamocortical burst also imposes an excitatory postsynaptic potential (EPSP) on pyramidal neurons, providing the basis for the spindle waveforms observed on scalp EEG.

### 2.2. Development of Sleep Spindles

In typically developing children, very early (“rudimentary”) spindles may be observed as early as term to 2 weeks postterm [15, 18–21] and it was proposed that they could appear earlier in premature infants [22]. Spindles become more easily identified between 3 and 9 weeks postterm, when they often occur in relatively long trains lasting up to 10 seconds during quiet sleep [21, 23, 24]. In the first 6 months, spindles may occur unilaterally, often alternating between hemispheres. Asymmetry is common as well. Spindles become increasingly more synchronous between hemispheres during the first year of life, reflecting maturation of interhemispheric connections [25]. By 12 to 18 months, most sleep spindles are expressed in a bilaterally synchronous and symmetric fashion, with maximal expression over the central regions.

Maturation alters sleep spindle activity in terms of the spindle frequency, amplitude, duration, and density [15, 18–20, 26, 27]. Changes in the development of spindle duration and density are thought to follow a U-shaped distribution, whereas that of the interspindle interval shows an inverted U-shape. Three distinct phases of sleep spindle development have been proposed: (1) infants up to 9-10 months old exhibit long spindles (around 1.5 s) having a relatively low density (<3/min) and a relatively short interspindle interval (around 20 s); (2) children from 10 months up to 3 years exhibit a decrease in the spindle length (to around 0.8 s) and an increase in the interspindle interval (up to 111 s), yielding an even lower spindle density (0.3–1.2/min); and (3) over three years of age, children show short interspindle intervals (5–10 s) and long spindle durations (0.9–1.5 s) of high density (4–10/min). These changes presumably reflect developmental changes in thalamocortical structures and maturation of the physiological system that produces spindles [26, 27]. Sleep spindle peak frequencies increase from childhood to adolescence [15, 28, 29], when global maturational changes in sigma power predominate in the slow sigma frequency band [29]. The topographic representation of sigma power provides insight into these age-related changes [30] by showing that the fast sigma power increases over the centroparietal areas, while the slow sigma power decreases over frontal areas across childhood and adolescence [15, 30]. Given the association between neural maturation and changes in sleep spindle activity, it has been proposed that sleep spindles could be used as a potential index of neural maturation [27].

### 2.3. Sleep Spindles and Cognitive Development in Typically Developing Children

Intellectual ability is closely related to cortical development in children and adolescents. Intelligence is associated with the trajectory of cortical development, primarily that of the frontal regions which are implicated in the maturation of intelligence [31, 32]. Synaptic density increases until around puberty (11 years for girls and 12 years for boys), whereupon synaptic pruning begins [33]. In fact, vigorous cortical thinning by early adolescence has a positive association with IQ [34].

A similar pattern is seen in the developmental changes of sleep spindles. Initially, during development, the increased neuronal connectivity results in higher sleep EEG amplitudes because the size of these waves reflects the number of synaptic connections [31, 35–37]. Thereafter, pruning results in smaller neuronal populations that oscillate in unison, with corresponding decreases in EEG power. These changes in the brain appear to parallel the rapid development of cognitive abilities at similar ages [31, 33, 38–43]. Synaptic pruning and increasing myelination during adolescence result in faster and more efficient information processing, which is manifested by an increased ability to perform complex cognitive operations, increased speed and efficiency in completing simple information-processing tasks, and improved performance on intelligence tests across adolescence [37].

Slow spindles have been correlated with visual perceptual learning [44], while fast spindles have been correlated with more complex abilities and processes, such as fluid intelligence [45], learning ability [46] and word-location associations [47]. It has been proposed that they could be used as a neurobiological indicator for the level of cognitive development.

### 2.4. Sleep Spindles and Cognitive Function in Typically Developing Children

The processes underlying sleep spindles have been hypothesized to benefit cognitive functions and “off-line” information processing in several ways. First, it is assumed that sleep spindles serve as a “gating mechanism” to protect sleep from being interrupted by external stimuli, such as noise [48], thereby allowing optimal time for off-line information processing. Historically, thalamocortical (TC) cells were thought to gate sensory transmission by switching from tonic to burst discharge mode [16]. The bursting pattern occurs during NREM sleep in the form of sleep spindles, while the tonic pattern of activity occurs in wakefulness. The tonic activity pattern was thought to relay sensory information to the cortex via the thalamus from a variety of afferent inputs, while the bursting or spindle pattern was thought to serve a gating role. More recently, researchers have proposed that both modes can relay stimuli to the cortex. However, while tonic spikes reliably transmit information, the stereotyped discharge profile of bursts leads to nonlinear distortion of sensory inputs [49]. Burst firing of TC cells during spindles would thus filter external stimuli. It has been proposed that one way in which sleep spindles could support cognitive function is by blocking interference (i.e., performing a gating function) to allow uninterrupted off-line processing and consolidation of information.

Sleep spindles appear to actively enhance information processing via their role in memory consolidation. Behavioral studies in animal models and humans have shown that learning improves more during and following a period of sleep than during an equivalent amount of waking time [50–52]. Neuroimaging studies have shown that the patterns of brain activity elicited during initial learning are replayed during subsequent sleep, demonstrating the presence of dynamic off-line information processing [53]. Underlying this enhancement or stabilization of memory across a sleep period is the concept of sleep-dependent memory consolidation, whereby memory traces become more stable and resistant to interference over a period of time. The consolidation of declarative memory results from a dialog between reactivated hippocampal memory traces and the neocortical networks that retain long-term memory representations [54]. In adults, sleep-dependent consolidation of declarative memory has been correlated with a range of neurophysiological measures, including the amounts of slow wave activity (1–4 Hz), slow oscillations (0.5–1 Hz), and sleep spindles. This indicates that sleep plays an active role in the hippocampal-neocortical dialog [54]. Recent research conducted in children of different ages has shown that their sleep-dependent consolidation of declarative memory is comparable to that of adults, whereas that of procedural skills is not [55, 56].

One explanation for how sleep spindles contribute to new learning is related to the hypothesis that sleep spindles facilitate the priming of synapses for plastic changes, [57] thereby enabling the off-line information processing that is essential for learning to be completed [58]. Data from simulations of sleep spindle activity have indicated that repetitive thalamic bursts (similar to sleep spindle activity) generate robust entry of Ca^2+^ into cortical dendrites [59], which produces conditions that favor the priming of synapses for plastic changes (i.e., by activating proplastic signaling molecules such as protein kinase A and Ca^2+^/calmodulin-dependent protein kinase II). The synaptic potentiation induced by spindles is consistent with the hypothesis of “active system consolidation” [60], which proposes that sleep spindles potentiate memory traces by reactivating selected neuronal circuits.

In addition to the contribution of sleep spindles to creating the conditions that allow for optimal sleep (i.e., by gating), brain plasticity, and memory consolidation, the characteristics of sleep spindles have been shown to be stable within subjects across different nights [6], and to correlate with intellectual ability, particularly fluid intelligence. This has led to the view that spindles, at least under non-pathological conditions, constitute to some extent a biophysical measure of intelligence.

In children, mixed results have been reported in regard to the direction and strength of associations between the different characteristics of sleep spindles and performance on intelligence tests and memory tasks. Geiger et al. found that peak sleep spindle frequency was negatively correlated with full scale IQ but that relative sigma power correlated positively with full scale IQ and fluid IQ [61]. Gruber et al. showed that lower sleep spindle frequency was associated with better performance on the intelligence perceptual reasoning and working memory WISC-IV scales but that sleep spindle amplitude, duration, and density were not associated with performance on IQ tests [62]. Chatburn et al. observed that the number of fast spindles was positively correlated with narrative memory and negatively correlated with sensorimotor functioning. Mean central frequency of spindles was also negatively correlated with sensorimotor functioning, planning ability, and working memory [63]. Hoedlmoser et al. found that children with higher sleep spindle activity, as measured at frontal, central, parietal, and occipital sites during both baseline and experimental nights, exhibited higher general cognitive abilities (WISC-IV) and declarative learning efficiency (i.e., number of recalled words before and after sleep) [64].

Astill et al. reported that individual differences in the dominant frequency of spindles and slow waves were predictive for performance on finger sequence tapping tasks. Children performed better if they had fewer slow spindles, more fast spindles, and faster slow waves. On the other hand, overnight enhancement of accuracy was most pronounced in children with more slow spindles and slower slow waves, that is, the children with an initial lower performance at baseline. Thus, associations of spindle and slow wave characteristics with initial performance may confound interpretation of their involvement in overnight enhancement. Slower frequencies of characteristic sleep events may be a marker of slower learning and immaturity of networks involved in motor skills [65]. Bódizs et al. found that fluid IQ correlated positively with fast spindle density and amplitude in girls and that these correlations peak in the frontocentral regions. In boys, by contrast, the only positive spindle-index of fluid IQ was found to be the frequency of fast spindles [66]. Doucette et al. observed that children with faster processing speeds exhibited higher slow sigma power over the parietal region [67]. For a detailed description of these findings in typically developing children please see Table 1.

In summary, sleep spindles appear in early infancy, and they change and develop through childhood and adolescence in a progression that parallels the milestones of cortical development. Sleep spindles appear to contribute to off-line information processing by protecting sleep (and thus sleep-dependent processes) from being interrupted. They also seem to facilitate the brain plasticity that allows essential learning to occur through processes such as memory consolidation. Finally, certain characteristics of sleep spindles have been correlated with intellectual performance, particularly fluid intelligence.

## 3. Sleep Spindles in Children with Neurodevelopmental Disorders

Despite the high prevalence of sleep problems in children with NDD and the wide range of cognitive deficits in this population, there is only limited information regarding the characteristics and function of sleep spindles in this population. Only a handful of studies have examined sleep in children with NDD, and the existing data are limited in scope and the range of disorders studied. The few existing studies on the putative association between sleep spindles and cognitive function have been conducted in children with intellectual disabilities, ASD, reading disabilities, and ADHD. However, although the body of knowledge is scattered, often based on nonstandardized methods, not always up-to-date, and based mostly on descriptive and correlational studies, it provides important insights into the extent to which sleep spindles are abnormal in these populations compared to typically developing children. Moreover, the existing studies give us an initial view of the nature and extent of the associations between sleep spindles and different intellectual levels in children with neurodevelopmental disorders or (in a few studies) a more direct idea of the associations between sleep spindles and information processing or intellectual performance.

### 3.1. Sleep Spindles in Children with Intellectual Disability

Intellectual disability is characterized by three findings: an intelligence quotient (IQ) of 75 or below; significant limitations in adaptive behaviors; and onset of disability occurring before age 18. In the past, the term “mental retardation” was used to describe this condition, but this term is no longer used [68]. The common causes of intellectual disability include genetic conditions, brain insults during pregnancy, problems at the time of birth, medical problems that affect brain health, and exposure to environmental toxins (e.g., lead or mercury).

Sleep problems are more severe and more prevalent in children with NDD than in typically developing children. Insomnia in normally developing young children is most often behaviorally based [69], whereas insomnia in children with NDD is more often multifactorial, with neurologic, medical, and psychiatric comorbidities contributing to behavioral issues. Moreover, insomnia in NDD tends to be chronic, often lasting into adolescence or adulthood [70]. Quine showed that sleep problems are persistent in children with developmental disabilities [71], and Wiggs and Stores found that the average duration of sleep problems in such children is 7.13 years (SD 4.04 years) [72].

Alarmingly high prevalence rates for sleep disorders have been cited in children with intellectual disabilities: 86% in children under 6 years old, 81% in those aged 6 to 11 years old [71], and 77% in those aged 12 to 16 years old [73]. Night waking and settling difficulties are particularly common, affecting over half of children with NDD aged up to 16 years. The chronicity of these problems was illustrated by Quine [71], who found that half of children with NDD with settling problems and over two-thirds of those with night waking were still having problems 3 years later.

Sleep disorders are also highly prevalent in children with ASD: recent studies have reported prevalence rates as high as 40–85% in these children versus 20–40% in typically developing children [74–76]. The commonly reported sleep disturbances include prolonged sleep-onset latency, restless sleep, frequent nocturnal awakenings, and reduced total sleep time. Fifty-three percent of 2- to 5-year-olds with ASD had at least one sleep problem, compared with 32% of controls [77]. Children with ASD often take more than 1 hour to fall asleep, and many have nocturnal awakenings that may last as long as 2-3 hours [78]. Sleep problems in ASD tend to persist past mid-puberty [79]. A longitudinal case-control study found that insomnia was 10 times more likely to be reported in ASD children than in controls, and that remission of sleep problems at 11 to 13 years was far less likely in ASD children than controls (8 versus 53%) [79]. The most common sleep problems in ASD are sleep-onset delay, frequent nocturnal awakenings, and reduced sleep duration [80].

As for ADHD, as many as 70% of children with this disorder have been reported to display mild to severe sleep problems [81]. The prevalence rates differ by ADHD subtype, with the highest prevalence in the combined subtype [82], though sleepiness may be more frequent in the inattentive subtype [83]. In addition, psychiatric comorbidities and medication used both increase the prevalence of sleep problems in ADHD [82]. Children and/or their parents reported bedtime resistance, sleep-onset difficulties, night awakenings, difficulties with morning awakenings, sleep breathing problems, and daytime sleepiness significantly more than healthy controls [84]. Although there is no sleep problem specific to ADHD [81], the most commonly reported issue is “difficulty falling asleep [81].”

#### 3.1.1. Sleep Spindle Characteristics in Children with Intellectual Disability

In 1962, E. L. Gibbs and F. A. Gibbs [85] reported the presence of “extreme spindles” in children with intellectual disability. Extreme spindles are characterized by their diffuse expression, much higher voltage (200–400 microvolts), and continuous occurrence. In addition, several studies examined sleep spindles in children with intellectual disability related to different genetic disorders [86]. These disorders included the neuronal ceroid lipofuscinosis (NCLs) [87] and Costello syndrome. The NCLs are a group of inherited progressive neurodegenerative lysosomal-storage disorders that are characterized by progressive intellectual and motor deterioration, seizures, and early death resulting from neural loss and widespread accumulation of lipopigments within cellular compartments [86]. The incidence (affected persons per live newborns) was reported to be 1 : 12,500 in the USA and Scandinavian countries, whereas the worldwide figure is 1 : 100,000 [88]. Children with NCLs were found to lack sleep spindles.

Costello syndrome, which is an autosomal dominant disorder caused by mutations in* HRAS*, has an estimated birth prevalence of 1 : 300,000 in the UK [89]. Costello Syndrome [90] is characterized by delayed development and intellectual disability, loose folds of skin, unusually flexible joints, and distinctive facial features including a large mouth. Researchers observed an increase in spindle amplitude (extreme spindles). Another study was conducted in children with malformations of cortical development [91]. These congenital brain disorders arise during embryonic and fetal development and are characterized by abnormalities in the volume, location, and/or architecture of cerebral gray and white matter. For each malformation subject, the authors identified a nonepileptic age- and sex-matched control patient for whom an EEG study had been performed within one year of the malformation subject's study, which was of the same type and demonstrated at least one sleep spindle burst. The authors found no difference between cases and controls in the mean spindle density or mean maximum spindle frequency, but significant between-group differences were seen in the laterality and anatomical distribution of spindles. Malformation subjects had a significantly higher proportion of unilateral sleep spindles compared to controls. Furthermore, although the sample sizes were small, subjects with unilateral malformations appeared to demonstrate a skewing of unilateral spindles toward the contralateral side, with fewer spindle bursts on the ipsilateral side to the malformation. Finally, malformation subjects had a significantly different overall anatomical distribution of sleep spindles, with an increased proportion of both anterior and diffuse spindles. Finally, Shibagaki et al. [92] reported a high prevalence of children with malformations of cortical development with no spindles, low incidence of sleep spindles, and/or extreme spindles.

#### 3.1.2. Sleep Spindles and Intellectual Performance in Children with Intellectual Disability

In a series of studies [92–94], Shibagaki et al. classified the participants' levels of functioning according to their developmental quotients (DQs). They used the Tsumori and Inage questionnaire for infants and children, which divides the children into severe, moderate, and mildly intellectually disabled groups. They included children with intellectual disability associated with a range of disorders (e.g., congenital cerebral dysplasia, hydrocephaly, Rubinstein-Taybi syndrome, Down's syndrome, and chromosomal abnormality) and performed several studies investigating the occurrences of sleep spindles in these children. They found that children with intellectual disability frequently have no sleep spindles and that these children tended to have a lower DQ than those with intellectual disability with sleep spindles [94]. They reported a significant increase in abnormal clinical EEGs and a significant decrease in the DQs of children with high occurrences of shorter or no sleep spindles compared to those with higher occurrences of longer sleep spindles [93, 95].

#### 3.1.3. Sleep Spindles and Memory Consolidation in Children with Intellectual Disability

No study has yet been reported that assessed memory consolidation in children with intellectual deficits.

Collectively, these studies show that children with intellectual disabilities due to a variety of heterogeneous genetic and developmental disorders show significant alterations in sleep spindles, including few or no sleep spindles, extreme spindles, and/or an increased proportion of unilateral spindles. In addition, the degree of sleep spindle abnormality is associated with the severity of cognitive impairment. In conditions with progressive neurodegeneration, spindle loss has been correlated with the state of the disease, with a complete absence of spindles seen during the most severe stages.

### 3.2. Sleep Spindles in Children with Autism Spectrum Disorders (ASDs)

ASDs are neurodevelopmental disorders characterized by repetitive behaviors and deficits in social interaction and communication. A diagnosis of ASD is made based on a constellation of requisite behavioral symptoms, including persistent deficits in social communication and interaction across multiple contexts, as well as restricted and repetitive patterns of behavior, interests, and activities.

#### 3.2.1. Sleep Spindle Characteristics in Children with ASD

Studies comparing children/adolescents with ASD versus controls showed that the former had a lower sleep spindle density (Godbout et al. [96]), fewer sleep spindles over the central regions [97], a lower NREM stage 2 sleep spindle density in the prefrontal area, and shorter sleep spindle duration in the frontal area [98]. However, another study failed to find any difference in sleep spindles when comparing children with ASD and typically developing children [99].

#### 3.2.2. Sleep Spindles and Intellectual Performance in Children with ASD

Reference [98] examined the associations between IQ (measured by the WISC-III) and sleep spindle density or duration in children with high functioning autism compared to typically developing children. The authors found that verbal IQ was negatively correlated with frontal spindle density and positively correlated with central spindle duration in typically developing children, whereas verbal IQ and the full scale IQ were negatively correlated with central spindle density in the ASD group.

#### 3.2.3. Sleep Spindles and Memory Consolidation in Children with ASD

One recent study [99] examined memory consolidation in children with ASD. Twenty-two participants with ASD and 20 control participants between 9 and 16 years of age were trained to criterion on a spatial declarative memory task and then given a cued recall test. The subjects were allowed a period of daytime wake (Wake) or a night of sleep (Sleep), both of which were monitored with home-based polysomnography. Upon retest, better memory consolidation was observed in the Sleep group compared to the Wake group for both ASD and control children; however, participants with ASD had poorer overall memory consolidation. The change in performance across sleep, independent of medication and age, showed no significant relationship with any specific sleep parameter other than the total sleep time, and there was a trend toward less forgetting in the control group.

In summary, there are conflicting findings with respect to sleep spindles in children with ASD. Whereas the data from one research group suggest that individuals with ASD have shorter spindle durations, lower spindle density, and decreased sleep spindle frequencies, other groups have not replicated these findings. In addition, children with ASD have poorer memory consolidation than controls.

### 3.3. Sleep Spindles in Children with ADHD

Attention deficit/hyperactivity disorder (ADHD) is one of the most common NDDs in childhood, affecting approximately 3%–5% of school-aged children [1] and enduring throughout adolescence and adulthood. A diagnosis of ADHD is dependent on developmentally inappropriate symptoms of inattention, hyperactivity, and/or impulsivity, with onset before the age of 7 years and impaired functioning in two or more settings [68].

#### 3.3.1. Sleep Spindle Characteristics in Children with ADHD

There are conflicting findings with respect to sleep spindle characteristics in children with ADHD. Some studies [100] failed to find any statistically significant difference in the number of sleep spindles between hyperactive and control children, while other studies found significantly fewer [101] or more [102] sleep spindles in the EEGs of unmedicated hyperactive boys compared to normal controls.

#### 3.3.2. Sleep Spindles and Intellectual Performance in Children with ADHD

No published study has yet examined this association.

At present, there is no consistent evidence for abnormal or altered sleep spindle activity in children with ADHD and sleep spindles activity per se has not been measured in studies examining memory consolidation of children with ADHD. However, there appear to be differences in the memory consolidation of children with ADHD versus typically developing children [103, 104], in that sleep benefits declarative memory in typically developing children, whereas ADHD children show deficits in sleep-associated consolidation of declarative memory and a reduced functionality of slow oscillations in this consolidation. In contrast, although procedural memory in typically developing children does not benefit from sleep, the data suggest that sleep appears to normalize the daytime deficits in procedural memory found among ADHD children [104].

### 3.4. Sleep Spindles in Children with Dyslexia

Developmental dyslexia is a hereditary neurological disorder that is characterized by the presence of severe and persistent reading and/or spelling impairments despite normal intelligence and adequate schooling. Almost no studies are available on sleep in children with dyslexia. The existing data have shown an alteration of sleep architecture characterized by an increase in slow wave sleep (SWS), a decrease in REM sleep, and a longer REM sleep latency in children with reading disabilities compared to controls [105].

One study [106] examined sleep spindles in 19 children with developmental dyslexia and 11 normally reading children between 7 and 16 years of age. The authors observed increases in spindle activity and sigma power in children with dyslexia and found that these parameters were correlated with the degree of dyslexic impairment. No information is available regarding sleep spindles and memory consolidation in this population. However, these results suggest that sleep spindle abnormalities may exist in children with dyslexia and that these abnormalities could be related to or correlated with impairment. Additional studies are needed to test these hypotheses.

## 4. Discussion

The goal of this paper is to review and integrate the available evidence regarding sleep spindle characteristics in children with NDD and (when possible) their associations with cognitive function. Before we attempt to integrate the findings across different disorders, we must note the significant methodological limitations of the existing work. Most studies had small sample sizes and thus might have overestimated the effect size and/or be difficult to replicate. This becomes even more challenging when researchers used data from the same participants in multiple publications (e.g., [94, 107]). Another limitation involves the heterogeneity of the samples. First, clinical heterogeneity is inherent with each of the NDD under discussion. In addition, many of the studies may have suffered from developmental heterogeneity, as they lumped together participants of different developmental stages in terms of their puberty, sleep, and cognition. Heterogeneity may also have arisen from the inclusion of participants having different intelligence levels and/or comorbid conditions. These sources of heterogeneity cause us to question whether the results could be generalized to other settings and situations. Additional issues are related to methodological differences between the studies, which make it difficult to compare results directly. For example, in some studies EEG patterns were recorded during a full night of sleep, while in others sleep was induced by chloral hydrate during the daytime and only sleep recordings from routine clinical EEG were analyzed. In the latter case, medication effect cannot be ruled out, and the amount of time spent in stage 2 NREM sleep was limited, meaning that fewer sleep spindles were detected.

These noted limitations may be inherent challenges of investigating sleep spindles and cognition in children with NDD. It could be difficult to obtain larger, more homogenous groups given the prevalence of the disorders and their clinical nature which includes significant comorbidity and diversity. Technically and financially, it is challenging to conduct laboratory-based sleep studies in children who are challenging to manage, often have difficulty tolerating electrodes, may dislike being in an unfamiliar environment, and can resist cognitive testing. Hence, practical issues pose real barriers for the feasibility of large, homogenous studies that use objective measures of sleep and cognition.

Our review integrates the existing data pertaining to sleep spindle characteristics in children with NDDs and examines the results from studies seeking to correlate these differences with cognitive processes. Table 1 presents the evidence we reviewed regarding sleep spindles in children with NDD and in typically developing children. Several studies have found lower spindle density [96–98, 108] and extreme spindles [96] in children with ASD. In children with intellectual disabilities, absence of sleep spindles [87, 93, 107], extreme spindles [85], increased spindle activity [90], unilateral sleep spindles [91], and higher ratio of children with long spindles [95] have been documented. In children with dyslexia, increase in power of frequency bands 0.5–3 Hz and 11-12 Hz during N2 have been found [106]. This observed phenotypic variability neither proves nor refutes the existence of shared mechanisms in NDDs. However, shared molecular mechanisms have been shown to operate across disorder boundaries. It has also been suggested that, in the future, NDDs may be defined as pathological deviation of specific developmental processes and/or be seen to represent stages on a continuum of neurodevelopmental causality [109, 110]. Although it has not been experimentally demonstrated, it is possible that alterations of sleep spindles may interfere with cognitive processes and behavior. Alternatively, it is possible that a proportion of neurodevelopmental impairments and sleep spindle alterations arise as independent manifestations of an underlying brain abnormality.

Even given the abovementioned issues, certain factors could be better controlled to meaningfully decrease the heterogeneity of the studies, thereby improving their relevance, validity, and generalizability. For example, better control of age and sex would allow future studies to focus on groups that are homogenous in these parameters. More studies might be required to cover all age groups for both sexes, but the information obtained in each study (even using small sample sizes) will be more significant. This will improve reproducibility and prevent findings which lack reliability.

### 4.1. Potential Mechanisms Underlying the Interplay between Sleep Spindles and Cognition in Children with Neurodevelopmental Disorders

One hypothesis regarding the nature of the association between sleep spindle characteristics and NDD is that impaired spindle activity could both reflect an abnormal neurodevelopmental trajectory and compromise the establishment of normal cognitive processes in this population. Although we do not yet know which pathways are involved or whether they are common to the various NDD, different hypotheses have been put forward regarding brain-related, genetic, and environmental influences. We will discuss these hypotheses in the context of the potential association between sleep spindles and NDD.

Many neurodevelopmental disorders (e.g., ADHD, ASD, and dyslexia) are accompanied by distinctive patterns of gray and white matter changes in the brain [111]. The evidence suggests that changes in gray matter may reflect structural changes in synapses and their dendrites, whereas those in the white matter reflect changes in myelination due to oligodendrocyte pathology. The presence of structural pathologies during development appears to provide a coherent biological model for the onset and course of NDD, while also suggesting a possible mechanistic basis for the associations between sleep spindles and cognitive abnormalities in such conditions [32, 112].

One factor that might facilitate the synchronization of neuronal networks and boost oscillatory activity is the strength of the underlying connections (i.e., the integrity of the white matter) [113, 114].

Individual differences in spindles and slow waves reportedly depend on the white matter microstructure across distributed networks. Thus, sleep oscillation profiles reflect both the synaptic-level dynamics of the neuronal network and the localized microstructural properties of the white matter tracts that form its structural backbone. Indeed, diffusion-tensor imaging revealed that the expression profiles of sleep slow waves and spindles are partially determined by the axial diffusivity strength over long-range white matter tracts [115]. In contrast, the higher-frequency waves (including the beta and gamma frequency bands) mostly reflect short-distance synchronization, which increases during early development and might involve short-range white matter axons rather than the long-range white matter tracts. Associations have been found between individual-level sleep spindling and white matter integrity [115] and continuing white matter development during late adolescence [116]. White matter abnormalities have been found in several NDD, including ADHD [117–122] and ASD. In the latter case, longitudinal data showed that white matter growth was slower among boys with autism, especially in the parietal lobes, [123] and diffusion imaging studies have revealed widespread disruption of white matter tracts in ASD patients, especially between regions implicated in social behavior [124–126]. Abnormal white matter development might, therefore, be a mechanism that cuts across neurodevelopmental disorders and might be related to both sleep spindles and disorders of cognitive function.

With respect to gray matter, the developmental changes in the prefrontal cortex differ depending on the IQ of the subject. The peak prefrontal gray matter volume is reportedly reached at 13 years in very high-IQ subjects, compared to 9 years in children of average IQ, with both groups reaching the same volume in late adolescence [32]. The growth and retraction of dendrites are dependent on the number and activity of the synapses that abut on their dendritic spines. Thus, changes in the gray matter volume are likely to reflect the formation and regression of synapses [127, 128]. The emergence of childhood ASD, ADHD, and dyslexia all involve divergence from the normal trajectories of gray matter development in different lobes of the brain (for review see [111]).

For sleep spindles, gray matter volume in the auditory and insular cortices was negatively correlated with sleep spindle frequency and found to be predictive of slower sleep spindles across individuals. Moreover, interindividual variability in sleep spindles over the sensory cortex was shown to predict the extent to which the sleep state is protected from experimental auditory stimulation [129]. These associations between slower sleep spindle frequency and gray matter volume in sensory areas may reflect the role of sleep spindles (of slower frequency) in protecting sleep against sensory disruption.

The association between sleep spindle frequency and the gray matter volume in the insular cortex provides a speculative basis for uniting various disparate findings in NDD. The loss of gray matter in NDD could result in abnormal sleep spindle activity, increasing the susceptibility to interference and thereby hindering sleep-dependent information processing. Future studies are needed to empirically test this possibility. Studies are also needed to examine the functional outcomes of abnormal white or gray matter volumes with respect to specific spindle characteristics, such as low frequency or higher density. Such associations could potentially explain a broad collection of cooccurring cognitive deficits in the NDD that are characterized by gray matter abnormalities (e.g., ASD, ADHD, and various learning disabilities).

### 4.2. Clinical Implications and Future Directions

This review indicates that future studies should continue examining sleep spindle characteristics and their associations with cognition in children with NDD. This is important because it is possible that aberrant spindle activity cuts across a number of physiological and pathological conditions, potentially reflecting impairments in neuroplasticity across these conditions. The examination of spindle measures in children with NDD could uncover developmental alterations that may characterize progression of these disorders. Furthermore, since sleep has been demonstrated to improve memory consolidation and learning, potential changes in sleep spindles may also be relevant to our complete understanding of the cognitive difficulties observed in many patients with NDD. Future studies should seek to use large, well-phenotyped samples that are homogeneous in age and sex and represent all developmental stages of the disorder being investigated. They should also use objective and standardized measures of sleep and cognition plus, when possible, experimental designs that will allow the authors to establish causality. Such studies would significantly advance our understanding of this topic and could move us toward answering important questions, such as what does it mean to have fewer or aberrant spindles, in terms of cognitive functioning? Can sleep spindle assessment in infancy and toddlerhood enable the early identification of learning or neurocognitive disabilities that could be addressed at an early age (e.g., ADHD or dyslexia), potentially reducing their negative impact on the developmental trajectory of at-risk children? Longitudinal studies examining sleep spindle characteristics and later outcomes in children at risk for NDD could begin to answer these questions and potentially identify reliable markers that could be used for routine sleep assessments of infants and toddlers. This could open the door to practical and relatively inexpensive methods for the early identification and prevention of potentially lifelong disorders that are debilitating yet treatable (e.g., dyslexia or ADHD).

Given the findings that implicate sleep spindles in memory consolidation and brain plasticity, another important question is whether it could be possible to stimulate sleep spindle activity as a means to improve cognition in children with NDD. Since little or no spindle activity is associated with poorer memory consolidation and/or intellectual performance, could the stimulation of sleep spindles or other oscillations during sleep increase brain plasticity, improve memory consolidation, and/or improve intellectual performance? One study conducted in children with ADHD provided promising initial evidence by suggesting that this may be the case; external enhancement of frontal slow oscillations (SO) at 0.75 Hz by transcranial oscillating direct current stimulation (DCS) was shown to elevate sleep-dependent memory in children with ADHD to the level of healthy controls. While children with ADHD showed worse memory performance than healthy controls when subjected to the sham condition, this memory deficit vanished following the application of DCS during sleep. Several other methods that have been shown to increase sleep spindle activity or slow wave activity during subsequent sleep have also improved sleep-dependent memory consolidation, including anodal transcranial DCS [130, 131], intensive physical exercise during the daytime [132], neurofeedback [133, 134], and pharmacological manipulation of spindle density [135]. Future studies should therefore investigate whether enhancement of sleep spindles through these or other means could improve sleep-dependent memory consolidation or other aspects of cognitive function in children with NDD.

In summary, relatively few studies have provided detailed examination of sleep spindle characteristics in children with NDD and their associations with cognitive function. Existing studies suffer from the use of multiple, often nonstandardized, methodologies and lack of exploration across the full range of the NDD from early to late stages. Despite these significant limitations, there is collective evidence that investigation of sleep spindles in children with and without NDD is important because the mechanisms that underlie spindle generation are involved with plasticity and with stabilization of sleep, both of which are known to be important in supporting optimal cognitive development and function.

## Figures and Tables

**(a):** 

Reference	Disorder	Cognitive impairment	Age	Sample size	Study design	Sleep measure		Cognitive measure (outcome/s)	Results		
						Recording parameters	Spindles scoring		Sleep	Cognition	Sleep and cognition
Kiesow and Surwillo, 1987 [100]	ADHD	None	ADHD patients: Range: 3–11 Mean: 6.69 Controls: Range: 3–11 Mean: 6.69	ADHD patients: 11 Controls: 11 100% males	Correlational study	10–20 International System of electrode placement	S2 sleep spindles		No significant differences		

Prehn-Kristensen et al., 2011 [103]	ADHD	None	ADHD: 12–16 Controls: 11–14	ADHD: 12 Controls: 12	Experimental Case-control Repeated Measures Design	PSG (sampling rate: 200 Hz with 12-bit resolution). EEG (C3, C4, A1, and A2), EOG, and EMG.	Sleep spindles detected using a band-pass filter of 12–14 Hz, of S2 sleep	Declarative memory consolidation task (recognition accuracy)	ADHD participants: more REM sleep and shorter SWS latency	(1) Overall reduced recognition accuracy in ADHD group. (2) Enhanced recognition accuracy in the sleep condition, but greater in control compared to ADHD	Positive association between (1) duration of non-REM sleep and (2) slow oscillation power and sleep-associated memory consolidation in healthy controls but not in ADHD

**(b):** 

Reference	Disorder	Cognitive impairment	Age	Sample size	Study design	Sleep measure			Cognitive measure (outcome/s)	Results		
						Recording parameters		Spindles scoring		Sleep	Cognition	Sleep and cognition
Godbout et al., 2000 [96]	ASD	None	AS: 7–53 Controls: 7–61	ASD: 8 Controls: 8	Correlational study	PSG		S2 sleep spindles: 12–15 Hz, lasting 0.5–2.0 s, no amplitude criteria		ASD participants: (1) More transitions from a waking epoch to REM sleep compared to healthy controls. (2) Made less transitions from a S2 epoch to REM sleep compared to healthy controls. (3) Lower S2 spindle density.		

Limoges et al., 2005 [108]	ASD	None	ASD: 16–27 Controls: 16–26	ASD: 16 Controls: 16	Correlational study	PSG. EEG (C3, C4, O1, and O2), and EOG, EMG.		S2 sleep spindles (C3, Fp1): 12–15 Hz, lasting 0.5–2.0 s, no amplitude criteria		ASD participants: (1) Increased time in S1 and decreased non-REM sleep and SWS (2) Lower S2 sleep spindle density (C3) compared to healthy controls		

Limoges et al., 2013 [97]	ASD	None	ASD: 16–27 Controls: 16–27	17 ASD 14 controls	Correlational study	PSG (C3, C4, O1, and O2), EOG, and EMG.		S2 sleep spindles	Sustained and selective attention (RT) working memory (span score) Declarative episodic memory (# of recalled figures) Sensory-motor procedural memory (contact time, # of trials) Procedural memory (time)	(1) Lower REM density. (2) Lower SWS% (3) Higher S1% in lower S2 spindle density (C3) in AS compared to controls	Poorer performance in ASD compared to controls on sustained attention working memory. Sensory-motor procedural memory	(1) Positive association in controls but not in ASD between the SWS% and declarative memory immediate recall (2) Negative associations in both groups between SWS% and learning the sensory-motor procedural memory task (3) Negative associations in ASD but not in healthy controls between S1% and declarative memory immediate recall; sensory-motor and cognitive procedural memory tasks and selective attention (4) S2 spindle density (C3) is negatively correlated with RT in the selective attention only in healthy controls (5) S2 spindle density (C3) negatively correlated with sensory-motor procedural memory during the learning phase only in ASD group

Maski et al., 2015 [99]	ASD	None	ASD and controls: 9–16 years	ASD: 22 Controls: 20	Experimental Case-Control Repeated Measures Design training session in the morning 30 minutes after habitual wake time after the night of sleep recording and a testing session in the evening	PSG. EEG (F1, F2, C3, Cz, C4, O1, and O2), EOG, and EMG.		Spectral power calculated for slow wave oscillation (0.5–1 Hz), delta (1–4 Hz), theta (4–7 Hz), alpha (8–11 Hz), sigma (12–15 Hz), and beta (16–20 Hz) frequency ranges. S2 sleep spindles (Cz): 12–15 Hz, lasting 0.5–2.0 s, no amplitude criteria	Sleep-dependent memory consolidation (difference in performance (in %) between training and testing phases)	Less REM sleep in ASD compared to control group	(1) In both conditions ASD group performed more poorly than control group (2) Both groups recalled better in the sleep condition compared to the wake condition	Slow oscillation power correlated with sleep dependent memory consolidation in the ASD group only

Tessier et al., 2015 [98]	ASD	None	ASD: 6–13 Controls: 7–12 Mean: 10.23 SD: 2.00	ASD: 13 Controls: 13	Correlational study	PSG. EEG (C3, C4, F3, F4, O1, and O2), EOG, and EMG.		S2 sleep spindles	Intelligence WISC-III. Performance IQ (PIQ), verbal IQ (VIQ), and full scale IQ (FSIQ) scores.	Lower S2 spindle density (Fp1) in ASD group compared to control group		(1) VIQ positively correlated with S2 spindle duration (C4) in control group but not in ASD group (2) VIQ is associated with spindle density only in ASD group (3) FSIQ is associated with spindle density only in ASD group

**(c):** 

Reference	Disorder	Cognitive impairment	Age	Sample size	Study design	Sleep measure		Cognitive measure (outcome/s)	Results		
						Recording parameters	Spindles scoring		Sleep	Cognition	Sleep and cognition
Bruni et al., 2009 [106]	DD	None	DD: 8–16 Controls: 7–16	DD: 16 Controls: 11	Correlational study	PPSG 10–20 International system	Power spectra calculated for delta, theta, alpha, sigma, and beta Sleep spindles Scored during N2.	(1) Memory and learning transfer (2) Reading test (speed and accuracy) (3) Writing test (accuracy) (4) Intelligence WISC-IIIR	Compared to healthy controls DD participants had the following: (1) Less stage-shifts per hour (2) Lower N2% (3) Less N3% (4) Increase in power of frequency bands 0.5–3 Hz and 11-12 Hz during N2 and 0.5–1 Hz during N3 (5) Increased spindle density	Mean IQ DD: FSIQ: 93.9 VIQ: 90 PIQ: 98.9	Only in DD group: (1) Sigma power band in N2 was positively correlated with the memory and learning transfer reading test reading test (2) Spindle density was positively correlated with performance on the word reading test

**(d):** 

Reference	Disorder	Cognitive impairment	Age	Sample size	Study design	Sleep measure			Cognitive measure (outcome/s)	Results		
						Recording parameters		Spindles scoring		Sleep	Cognition	Sleep and cognition
Marca et al., 2011 [90]	ID	Costello syndrome (CS)	CS patients: Range: 18 months–31 years Controls: 18 months–31 years	CS: 11 Controls: 22				Sleep Spindles: 12–14 Hz. Scored over all NREM stages	DQ IQ	CS group had increased spindle activity between 13 Hz and 14 Hz and between 14 Hz and 15 Hz compared to healthy controls		

Selvitelli et al., 2009 [91]	ID	Malformations of cortical development (MCDs)	MCD Mean: 35.7 SD: 12.2 Controls: Mean: 35.6 SD: 14.3	MCD: 10 Controls: 10	Correlational study		Not specified	Sleep spindles; 12–16 Hz. Occurring during S2.		Compared to control group, MCD group had (1) higher proportion of unilateral sleep spindles (2) an increased proportion of anterior and diffuse spindles		

Shibagaki et al., 1982 [94]	ID	Congenital cerebral dysplasia (*N* = 10) Hydrocephaly (*N* = 3) Rubinstein-Taybi syndrome (*N* = 2) Down's syndrome (*N* = 1) Silver's syndrome (*N* = 1) Lesch-Nyhan syndrome (*N* = 1) Hallermann-Streiff syndrome (*N* = 1) Remaining subjects = unknown etiology	4 months–8 years	45	Descriptive study	PSG. EEG (F3-C3, F4-C4, F3-A1, F4-A2, C3-A1, C4-A2, O1-A1, and O2-A2), EOG, EMG, and ECG		Not specified	DQ IQ	(1) 56% displayed REM burst during NREM with sleep spindles. (2) 64% showed sleep spindles at the beginning or towards the end of REM sleep		No significant findings

Shibagaki and Kiyono, 1983 [95]	ID	Down's syndrome (*N* = 3) Hydrocephaly (*N* = 9) Holoprosencephaly (*N* = 2) Remaining subjects = unknown etiology	3–8 months	90	Descriptive study	PSG. EEG (F3-C3, F4-C4, F3-A1, F4-A2, C3-A1, C4-A2, O1-A1, and O2-A2), EOG, EMG, and ECG		S2 sleep spindles	DQ IQ	(1) 42% had a ratio of more than 2.00 (# spindles longer than 0.4** **s/# of spindles shorter than 0.4** **s) (2) 15.6% had a ratio of 1.99–1.00 and 6% had a ratio of 0.99–0.50 (3) 22% had a ratio of less than 0.50 (4) 14% had no sleep spindles		(1) Subjects with a ratio of less than 0.50 had significantly lower DQs than subjects with a ratio of more than 2.00 (2) Subjects with no sleep spindles at all had significantly lower DQs than subjects with a ratio of more than 2.00 (3) Subjects with a ratio of less than 0.50 or no sleep spindles had significantly lower DQs than subjects with a ratio of 1.99–1.00

Shibagaki, et al., 1986 [92]	ID	Cerebral palsy (CP) (*n* = 23)	CP: 4 months–5 years Non-CP patients: 4 months–12 years	CP: 23 Non-CP patients: 39	Descriptive study	PSG (F3-A1, C3-A1, and O1-A1), EOG, ECG, respiration, and EMG		Not specified	DQ IQ	(1) 13% of non-CP patients had no spindles or extremely low incidence of spindles (2) 8% of CP cases showed no sleep spindles (3) 22% of CP cases showed extremely low incidence of spindles (4) 4.3% of CP cases showed extreme spindles		CP patients with indistinguishable sleep stages had lower DQs than CP patients with normal EEG patterns and non-CP patients with normal EEG patterns

Shibagaki, et al., 1980 [93]	ID	Holoprosencephaly (*N* = 1) Down's syndrome (*N* = 2) Hydrocephaly (*N* = 8) Rubinstein-Taybi syndrome (*N* = 2) Prader-Willi syndrome (*N* = 2) Cri-du-chat syndrome (*N* = 2) Hallermann-Streiff syndrome (*N* = 1) Silver's syndrome (*N* = 1) Remaining subjects - unknown etiology	4 months–8 years	43	Descriptive study	PSG. EEG (F3-C3, F4-C4, F3-A1, C3-A1, F4-A2, and C4-A2), EOG, ECG, respiration, and EMG		Spindles measured during S2. Extreme spindles: 9-10 c/s, 50–120 *μ*V	DQ	(1) 2.3% had no REM sleep (2) 30% had no sleep spindles (3) 4.6% had high voltage fast activity (20–30 c/s, 100–200 uV) without sleep spindles in wakefulness, S1, S2, and REM (4) 7% had low voltage activity without spindles throughout nocturnal sleep (5) 2.3% had indistinguishable stages with the presence of sleep spindles (6) 2.3% had extreme spindles		(1) Patients with abnormal EEG patterns throughout S1–S4 had lower DQs than patients with abnormal EEG patterns only in S1-S2 and patients with normal EEG patterns (2) Patients with abnormal EEG patterns in S1-S2 had lower DQs than patients with normal EEG patterns

Veneselli et al., 2001 [87]	ID	Late infantile neuronal ceroid lipofuscinosis (LINCL)	Range: 3–10	LINCL patients: 18	Descriptive study	PSG. EEG (10–20 International System of electrode placement)		Not specified		No sleep spindles were observed		

**(e):** 

Reference	Disorder	Cognitive impairment	Age	Sample size	Study design	Sleep measure		Cognitive measure (outcome/s)	Results		
						Recording parameters	Spindles scoring		Sleep	Cognition	Sleep and cognition
Astill et al., 2014 [65]	None (TPD)	None	Mean: 10.7 SD: 0.8	30	Experimental Repeated Measures Design	PSG EEG (Fpz, Cz), EOG, EMG	Artifact-free EEG across S2, S3 and S4 Fast spindles: frequency ≥ 12 Hz, Slow spindles: frequency <12 Hz.	Sleep-dependent procedural memory task: finger sequence tapping tasks (speed and accuracy)	Average duration of slow waves at FPz was negatively correlated with density of fast spindles and positively correlated with density of slow	Accuracy was better if the interval contained sleep	(1) Higher SWS% was associated with increases accuracy (2) Children with higher density of slow spindles had lower overall speed and accuracy whereas children with higher density of fast spindles had higher overall speed (3) Children with higher density of slow spindles showed a stronger overnight increase in accuracy but not speed. (4) Children with a longer average duration of slow waves had stronger overnight increase in accuracy but not speed.

Bódizs et al., 2014 [66]	None (TPD)	None	15–22 years	24	Correlational study	PSG 10–20 International system	Measured across stages S2–S4.	Fluid intelligence Raven's progressive matrices test (RPMT) (IQ)	Subjects had normal sleep structure	IQ	Only in females, fast spindle density and fast spindle amplitude were positively correlated with IQ scores.

Chatburn et al., 2013 [63]	None (TPD)	None	4.1–12.7 years	27	Correlational study	PSG (C3-A2 and C4-A1), EMG, and EOG	S2 sleep spindles	Stanford-Binet intelligence scale NEPSY	Subjects had normal sleep structure	All intelligence and neurocognitive functioning was in the normal range	(1) Mean central spindle frequency was negatively correlated with nonverbal working memory, planning, and fine motor functioning (2) Fast spindle density was positively correlated with narrative memory and negatively correlated with sensorimotor functioning and fine motor functioning

Doucette et al., 2015 [67]	None (TPD)	None	2–5 years	10 50% males	Correlational study Sleep was recorded over 1 night in the home environment	PSG	Slow sigma frequency range: 10–13 Hz Fast sigma frequency range: 13.25–17 Hz	Processing speed task: (RT)	Subjects had normal sleep structure	Average reaction time was 1408.8 ± 251.4 ms	RT significantly correlated with slow sigma power in a cluster of 16 electrodes in parietal regions

Geiger et al., 2011 [61]	None (TPD)	None	Range: 9–12 years Mean: 10.5 SD: 1.0	14 57.1% males	Correlational study Sleep was recorded over 2 nights separated by 1 or 2 weeks in a sleep laboratory	PSG. EEG 10–20 International system EOG and EMG	Mean all-night power spectra of stage N2 sleep.	Intelligence WISC-IV Attention task (RT)	Subjects had normal sleep structure	All results were in the normal range	(1) Sleep spindle peak frequency correlated negatively with full scale IQ (2) Individual relative sigma power correlated positively with full scale IQ and fluid IQ

Gruber et al., 2013 [62]	None (TPD)	None	Range: 7–11 years	29	Correlational study	PSG (F3, F4, C3, C4, P3, P4, O1, and O2), EOG, ECG, and EMG	Measured during all NREM artifact-free epochs.	Intelligence WISC-IV	Subjects had normal sleep structure	All results were in the normal range	Sleep spindle frequency was negatively associated with scores on the perceptual reasoning and working memory

Hoedlmoser et al., 2014 [64]	None (TPD)	None	Range: 8–11 years	54	Correlational study	PSG 10–20 International system	Sleep spindles measured during N2 and N3.	Declarative memory consolidation task: word pair association task (RT) Intelligence WISC-IV	Subjects had normal sleep structure	(1) RTs for correctly remembered word pairs improved overnight (2) Poorer retrieval scores observed in the morning after sleep	(1) Higher SpA positively correlated with better memory performance overall before and after sleep (2) Children with higher IQ had higher SpA

*Note*. AASM = American Academy of Sleep Medicine; ADHD = attention deficit hyperactivity disorder; AS = Asperger's syndrome; ASD = autism spectrum disorder; CP = cerebral palsy; CS = Costello syndrome; DD = developmental dyslexia; DQ = developmental quotient; ECG = electrocardiogram; EEG = electroencephalogram; EOG = electrooculogram; EMG = electromyogram; ID = intellectual disability; IQ = intelligence quotient; KITAP = test of attentional performance for children; MCD = malformations of cortical development; NEPSY = neuropsychological assessment; PSG = polysomnography; REM = rapid eye movement; RT = reaction time; SD = standard deviation; SpA = spindle activity; SWS = slow wave sleep; TDP = typically developing children; WISC = Wechsler intelligence scale for children.
